# Analysis of the Frequency of the A1 and A2 Alleles in the Beta-Casein Gene and the A, B and E Alleles in the Kappa-Casein Gene in Local Cattle Breeds: Polish Red and Polish White-Backed

**DOI:** 10.3390/ijms26052212

**Published:** 2025-02-28

**Authors:** Wioletta Sawicka-Zugaj, Witold Chabuz, Joanna Barłowska, Sebastian Mucha, Karolina Kasprzak-Filipek, Agnieszka Nowosielska

**Affiliations:** 1 Department of Cattle Breeding and Genetic Resources Conservation, University of Life Sciences in Lublin, 20-950 Lublin, Poland; wioletta.sawicka@up.lublin.pl (W.S.-Z.); witold.chabuz@up.lublin.pl (W.C.); karolina.kasprzak-filipek@up.lublin.pl (K.K.-F.); 2 Department of Quality Assessment and Processing of Animal Products, University of Life Sciences in Lublin, 20-950 Lublin, Poland; 3 Polish Federation of Cattle Breeders and Dairy Farmers in Warsaw, 00-515 Warsaw, Poland; s.mucha@pfhb.pl (S.M.); a.nowosielska@pfhb.pl (A.N.)

**Keywords:** beta-casein (*CSN2*), kappa-casein (*CSN3*), local cattle breeds, Polish White-Backed, Polish Red

## Abstract

In view of the threat to local breeds resulting from intensive animal production, many studies are conducted in search of arguments confirming their importance in food production. In the case of milk production, not only is its quantity important, but its quality is as well, including its chemical composition. Particular focus has recently been placed on the casein proteins beta-casein (*CSN2*) and kappa-casein (*CSN3*), due to their potential impact on human health or on the suitability of milk for cheese production. The present study analysed the polymorphism of these proteins in 1777 cows belonging to two local cattle breeds, Polish Red and Polish White-Backed, using Illumina Infinium XT SNP technology on a EuroGenomics MD chip. The results indicate that the Polish White-Backed breed is predisposed to produce ‘A2 milk’, as the frequency of the *CSN2* A2 allele in the population was 61.2%. The Polish Red breed was characterised by a higher frequency of the *CNS3* B allele (35%), which according to extensive scientific literature is associated with better coagulation properties, and increased whey expulsion. The highest yield of milk and its constituents, confirmed at *p* ≤ 0.01, was obtained for Polish White-Backed cows with the A2A2 genotype in *CSN2* and cows with the AA genotype in *CSN3*. In the Polish Red breed, no statistically significant differences were obtained between means for milk production traits.

## 1. Introduction

In the last few decades, owing to the intensification of production systems, breeding selection, and modern technology, such as artificial insemination and genomic selection, the production of both meat and dairy cattle has doubled [1,2]. This intensification is meant to provide benefits in the form of greater food accessibility, but it increasingly arouses controversy regarding negative effects on the environment, animal welfare, or genetic diversity in cattle [3,4,5,6,7]. In the context of threats to the biodiversity of cattle, special attention should be focused on local breeds, of which 1027 are currently being raised worldwide. These breeds account for 83.16% of all cattle breeds, with the highest number found in Europe—362 [8]. It is worth noting, however, that the high breed diversity of this group does not correspond to the size of its individual populations. For many years, the most popular and most numerous cattle breed in the world, according to FAO [8], has been Holstein-Friesian, present in 135 countries. In Poland, this breed accounted for nearly 90% of cows used for dairy purposes at the end of 2023 [9].

The widely discussed topic of threats to biodiversity at all levels of the organisation of nature has focused attention on farm animals and raised great interest in native breeds. However, due to their low productivity compared to highly productive breeds, the stability of their future may rely on their use for the production of regional, niche products. Many examples of regional products are known, especially cheeses, which are popular not only in their region of production but often all over the world. These products, through certification and promotion, increase the profitability of raising local breeds, thus protecting them against extinction [10,11]. An excellent example is the indigenous Italian cattle breed Reggiana, whose milk was used to produce Parmigiano-Reggiano cheese as early as the 13th century, in the abbeys of Benedictine monks. However, the appearance of cosmopolitan, highly productive breeds in the second half of the 20th century threatened the popularity of the Reggiana breed, and the size of the population fell to just 450 in the 1980s. To prevent the extinction of the Reggiana breed, a programme for its conservation was initiated in the 1990s. In addition, the National Association of Reggiana Cattle Breeders created an additional brand for Parmigiano-Reggiano cheese (ChNP)—Parmigiano Reggiano delle Vacche Rosse, produced exclusively from the milk of this breed. Consumers’ growing interest in the cheese led to an increase in the demand for milk from the Reggiana breed and thus to a reversal of the downward trend in the population. The significant increase in the number of these animals to 4752 in 2023 can be regarded as a successful outcome of these measures [12,13,14].

**Figure 1:**
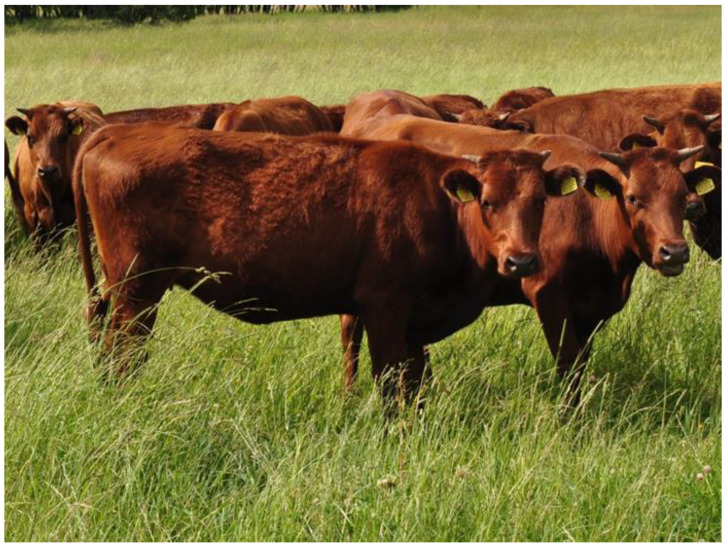
Polish Red females.

**Figure 2:**
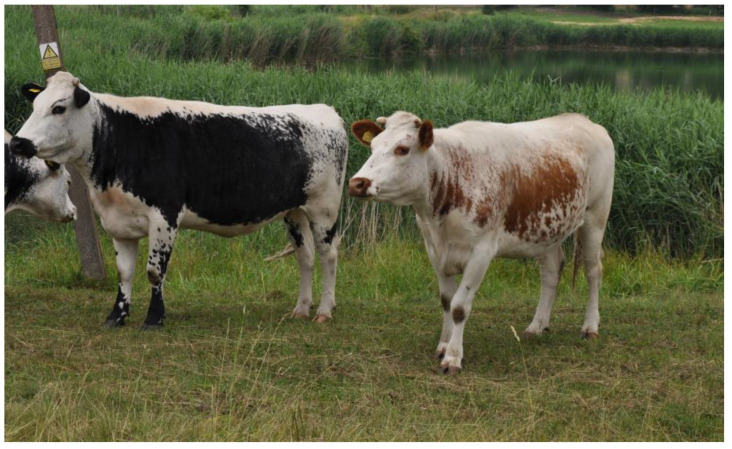
Polish White-Backed females.

In Poland, among the four cattle breeds included in genetic resources conservation programmes, two stand out: Polish Red and Polish White-Backed. The former, classified as brachyceros, is distinguished by a solid red coat colour (Figure 1) and is concentrated mainly in the south of the country (where it is used for dairy purposes) and in the north (where it is raised for meat) [15]. Polish White-Backed cattle, classified as primigenius, have a characteristic colouring with a white stripe on the back (Figure 2). Their range encompasses the entire country, although the highest numbers are kept in the east [16]. Both of these breeds are indigenous and dual-purpose but used mainly for dairy production.

The quality of the final dairy product is determined primarily by the quality of raw milk, including its proportions of chemical components. In the context of cheese production, proteins and their individual fractions are crucial. The most commonly analysed proteins are casein proteins, αS1-casein (*CSN1S1*), β-casein (*CSN2*), and κ-casein (*CSN3*), responsible for rennet coagulation of milk and for cheese yield and quality [17,18,19]. Particular focus is placed on locus *CSN3*, located on chromosome 6q31, which has five exons and four introns, with most of the sequences coding the protein located on exon 4 [20]. In the *CSN3* gene, 14 allele variants have been identified (A, AI, B, B2, C, D, E, F1, F2, G1, G2, H, I, and J), encoding 13 proteins and 1 synonym (AI) [19]. The most common variants in cattle are A and B [21], at amino acid positions 136 and 148 of the primary structure [22]. Many years of research have shown that the *CSN3* B allele is favourably associated with milk coagulation parameters and curd quality [23,24]. In addition, milk from cows with the *CSN3* BB and AB genotypes is distinguished by faster rennet clotting time, better curd firmness, and higher cheese yield [25,26]. As milk coagulation parameters are extremely important for the dairy industry, bulls used for artificial insemination are genotyped for *CSN3*, and this is often a selection criterion in breeding [23,27].

In the context of milk proteins and, in particular, their connections with human health, there has recently been great interest in β-casein (*CSN2*), especially the A2A2 genotype [28,29,30,31]. *CSN2* accounts for up to 45% of casein in cow’s milk and is also located on chromosome 6 [20]. The primary structure of the gene was first described by Ribadeau-Dumas [32]. Further analyses showed the presence of 12 genetic variants (A1, A2, A3, B, C, D, E, F, G, H1, H2, and I) [33], among which A1 and A2 are the most frequent [28,34]. It is worth noting that the original form of *CSN2* is the A2 allele, and the A1 variant was the result of mutation [35]. They differ in one amino acid at position 67, with proline in A2 and histidine in A1. This change causes a cleavage during digestion and the release of the bioactive peptide beta-casomorphin (BCM7) [36], which according to ul Haq et al. [37] may be responsible for chronic inflammatory reactions, such as allergies, mucin production, lymphocyte proliferation, and skin reactions. Moreover, a study by Kamiński et al. [38] showed that milk with the A1 protein produces four times as much BCM-7 as A2 milk, which may potentially be linked to serious health problems such as type 1 diabetes, heart disease, atherosclerosis, sudden infant death syndrome, autism, and schizophrenia [39,40,41,42].

Given the role of the *CSN2* and *CSN3* proteins in dairy production, the aim of the study was to analyse these milk proteins for the presence of the A2A2 genotype in *CSN2* and the AB and BB genotypes in *CSN3* in two local cattle breeds: Polish White-Backed and Polish Red.

## 2. Results

Five variants of casein proteins were identified in the populations of Polish Red and Polish White-Backed cows, i.e., *CSN2* A1 and A2 and *CSN3* A, B, and E (Table 1). Four of these (A1, A2 in *CSN2* and A, B in *CSN3*) were present with a frequency of over 0.50. In the case of *CSN2*, there were major differences in the frequency of alleles between breeds. The A1 allele was the most common in the Polish Red breed (0.570), and A2 in Polish White-Backed (0.612). In the case of the *CSN3* gene, the distribution of allele frequencies was similar in both breeds, i.e., the A allele was the most common (0.635 in Polish Red and 0.669 in Polish White-Backed) and the E allele was the least common (0.015 in Polish Red and 0.018 in Polish White-Backed). On the other hand, the B allele occurred with higher frequency in the Polish Red breed (0.350) than in the Polish White-Backed breed (0.313). The χ^2^ values calculated for the *CSN2* and *CSN3* genes for both breeds indicate Hardy–Weinberg equilibrium in the populations. In *CSN2*, χ^2^ = 0.00 for Polish Red and 1.90 for Polish White-Backed (d.f. = 1; *p* = 0.98 and *p* = 0.17, respectively), and in *CSN3* χ^2^ = 1.98 for Polish Red (*p* = 0.58; df = 1) and χ^2^ = 5.84 for Polish White-Backed (*p* = 0.12; df = 1).

The analyses of genetic indices showed that the observed number of alleles in both breeds was the same in *CSN2* (two alleles) and in *CSN3* (three alleles), while the effective number in both genes was higher in the Polish Red breed—1.962 in *CSN2* and 1.900 in *CSN3*. Both breeds showed low heterozygosity, both observed and expected (<0.5), in both *CNS2* and *CNS3*. However, the Polish Red breed had higher levels of H_O_ and H_E_ in *CSN2* (0.490) and in *CSN3* (0.474 and 0.479) than the Polish White-Backed breed. Moreover, in the Polish Red breed, the observed heterozygosity in *CNS2* was equal to the expected heterozygosity, determined by the fixation index (Fis) of 0.000 (Table 2). In the same breed, heterozygote excess was noted in *CNS3* (Fis = −0.011), as the expected heterozygosity (0.479) was higher than the observed heterozygosity (0.474). In the Polish White-Backed breed, slight heterozygote excess was noted in both *CNS2* and *CNS3*, with Fis equal to 0.049 and 0.064, respectively. The F statistic provided information on the distribution of genetic variation between the cattle breeds based on the frequencies of alleles identified in the *CSN2* and *CSN3* genes. For the Polish Red breed, a negative Fit value (−0.011) was obtained for *CSN3* and a value of 0.00 for *CSN2*. The 0.000 Fst values obtained for the two breeds indicate a lack of differentiation between the populations.

The analysis of the frequency of haplotypes in the *CSN2_7*, *CSN3_AY380228_13068*, and *CSN3_AY380228_13124* genes in the Polish Red and White-Backed breeds revealed the absence of the A1-A-E and A2-B-E haplotypes (Table 3). Among those present, in Polish Red, the frequency of haplotype A1-B-E was the lowest (0.015), and that of A1-B-A was the highest (0.354). The frequency of the remaining haplotypes ranged from 0.149 (A2-A-A) to 0.281 (A2-B-A). It is worth noting that the A2-B-A haplotype in Polish White-Backed was the most frequent in the population (0.495), while the A1-B-E haplotype, as in the Polish Red breed, was the least common (0.015). The frequency of the remaining haplotypes ranged from 0.115 (A2-A-A) to 0.196 (A1-A-A).

The results of analysis of the frequency of haplotypes in the *CSN2* and *CSN3* genes, i.e., the absence of A1-A-E and A2-B-E and the very low frequency of A1-B-E, are reflected in the assessment of linkage disequilibrium (LD) in both cattle populations. The low LD values, between 0.01 and 0.11, presented in Table 4 indicate the lack of linkage between the genes analysed in both the Polish White-Backed and Polish Red breeds.

Table 5 presents the frequencies of *CSN2* and *CSN3* genotypes in the cattle breeds. In the case of *CSN2*, the most common genotype in both Polish Red and Polish White-Backed cattle was A1A2 (0.490 and 0.452, respectively). In the case of homozygous genotypes, A2A2 was predominant in Polish White-Backed cows (0.386) and A1A1 in Polish Red (0.325). Within *CSN3*, the most common genotypes in both breeds were AA and AB. In the Polish Red breed, 0.450 of cows had the AB genotype and 0.399 had the AA genotype. The reverse pattern was observed in Polish White-Backed cows, with a higher frequency of the AA genotype (0.462) than the AB genotype (0.391). The frequency of BB was similar in both breeds—0.122 in Polish Red and 0.112 in Polish White-Backed. The AE and BE genotypes were very rare in both breeds, and the EE genotype was only present in Polish White-Backed (0.002).

Table 6 and Table 7 present the yield and chemical composition of milk from Polish Red and Polish White-Backed cows. It should be noted that although the duration of the first lactation differed only slightly between the breeds, by only 4.96 days, White-Backed cows produced 403.45 kg more milk. On the other hand, the milk of Polish Red cows had higher contents of fat (4.37%), casein (2.61%), lactose (4.76%), and dry matter as well (13.13%). The analysis of the milk performance parameters of Polish Red cows in relation to different *CSN2* and *CSN3* genotypes showed no statistically significant differences between means. However, slightly higher yields of milk and its basic constituents were noted for the A1A2 genotype in *CSN2* and the AE genotype in *CSN3*.

In the case of the White-Backed breed, cows with the A2A2 genotype in *CSN2* had the highest yield of milk (4093.43 kg), fat (166.91 kg), protein (135.37 kg), casein (105.72 kg), lactose (192.90 kg), and dry matter (524.88 kg). These results were confirmed statistically (*p* ≤ 0.01). The percentage content of individual milk components was very similar for all three genotypes. In the case of the *CSN3* gene, the highest yield of milk (4212.09 kg), fat (171.77 kg), protein (138.86 kg), casein (108.30 kg), lactose (198.28 kg), and dry matter (539.04 kg) was observed for the most common genotype in this breed group, AA. These results were confirmed statistically (*p* ≤ 0.01). Interestingly, milk from cows with the BE genotype had the highest percentage content of all chemical components of milk (fat—4.18%, protein—3.44%, casein—2.67%, lactose—4.73%, and dry matter—13.00%), but these differences were not confirmed statistically.

## 3. Discussion

This study presents a description of the two main milk casein proteins in indigenous Polish cattle breeds. To the best of our knowledge, this is the first study to present this type of results on the basis of SNPs in the breeds Polish Red and Polish White-Backed. Indigenous breeds are a subject of great interest among scientists because they are an important reservoir of genetic variation in farm animals, and furthermore, the material they produce often has a unique composition and quality, which can be used to produce niche food products [44].

The study focused on two types of casein, β (*CSN2*) and κ (*CSN3*), for which the world literature shows numerous connections to human health or suitability for processing [27,29,45]. In the case of *CSN2*, high consumer awareness and increasing attention to a proper diet has raised a great deal of interest in ‘A2 milk’, obtained from cows with the A2A2 genotype [46,47,48]. This has prompted the dairy industry to respond to the public’s new demands by producing A2 milk in suitably selected cow herds [47], and consumers, as shown in a survey study by Fernández-Rico et al. [48], are willing to purchase A2 milk even at a higher cost than that of conventional milk. In the present study, the Polish White-Backed breed showed a high frequency of the A2 allele (0.612). A similar frequency was reported by Miluchová et al. [36] in Holstein cattle in Slovakia (0.632). Sanchez et al. [24] reported a very high frequency of the A2 allele in cattle breeds kept in France—Abondance (0.828), Brown Swiss (0.787), and Jersey (0.766), while Kulibaba et al. [49] demonstrated a frequency of 0.78 in Holstein-Friesian cows in Ukraine, and Kamiński et al. [50] reported a frequency of 0.590 in Holstein-Friesian cows in Poland. Studies by many authors [51,52,53,54] have shown that in breeds belonging to the *B. indicus* group from Asia and Africa, e.g., Dangi, Gir, Deoni, Khiller, and Surti, only the A2 allele occurs in *CSN2*. It is also present in the local Greek breeds Katerinis and Sykias [55]. Only this variant is also confirmed to be present in the water buffalo population [54,55,56]. In the Polish Red breed, the frequency of the *CSN2* A2 allele obtained in the present study was similar to the value reported by Cieślińska et al. [57] (47%), although that value was obtained using the PCR-ACRS (amplification-created restriction site) method. While the results of the present study indicate the potential of local Polish breeds to produce ‘A2 milk’, it is worth noting the effect of the A2 allelic variant in *CSN2* on characteristics associated with cheese production. Unfortunately, numerous studies [29,58,59,60] indicate that its presence in milk is associated with longer rennet clotting time, longer curd formation time, reduced curd firmness, and lower cheese yield compared to the A1 variant.

In *CSN3*, the A allele appeared with the highest frequency, in both the Polish White-Backed and Polish Red breeds. The frequency of this allele was similar in German breeds (0.644) [61], in Czech Fleckvieh cows (0.649) [62], and in the Swedish Red Polled and Swedish Red breeds (0.625 and 0.655, respectively) [63]. A study in Holstein-Friesian cattle kept in Macedonia also showed a higher frequency of the A allele (0.584) compared to the B allele (0.336), as well as the presence of the allele E at a level of 0.080 [64]. In Holstein-Friesian cows in Poland, Kamiński et al. [50] also demonstrated a higher frequency of the A variant (0.470) than the B variant (0.420), but the difference was not great. Chessa et al. [65] reported clear dominance of the B allele (0.90) in Jersey cows raised in Italy.

Interestingly, Hassan et al. [66], in their analysis of the milk of three Sudanese *B. indicus* cattle breeds, showed an even higher frequency of the *CSN3* A allele (0.817 in Butana cattle, 0.771 in Kenana cattle, and 0.875 in crosses of Kenana or Butana with Friesian). However, the B allele, in the BB or AB genotype, is widely believed to be the most suitable for cheese production [67,68,69]. In the present study, these genotypes were shown to have a similar frequency in the breeds analysed, although the AB genotype was slightly more common in the Polish Red breed than in the Polish White-Backed breed. The higher frequency of the BB and AB genotypes in *CSN3* in the Polish Red breed is consistent with 20 years of research on the suitability of milk from a large population of local Polish cattle breeds for cheese-making [70,71,72,73]. Those studies, initially carried out using Schern’s method and then with a Lactodynamograph, clearly showed that the milk of Polish Red cows had the most favourable parameters, i.e., the shortest clotting time and curd formation time and the firmest curd structure after 30 min. Milk from Polish White-Backed cows had similar parameters to those of milk from the Simmental breed, while the poorest results were obtained for Polish Holstein-Friesians [71]. Although the *CSN3* B allele is generally much less common than the A allele [74,75,76], Pazzola et al. [27] showed monomorphism of the *CSN3* B allele in the Italian breed Sardo-Modicana, whose milk is used to produce the well-known Pecorino cheese. However, numerous studies on the milk performance of cows have shown that in addition to genetic factors, environmental factors are extremely important. These include diet [77], housing system [78], stage of lactation [79], and health [80]. The confirmation of the presence of the AB genotype in *CSN3* at a level of 0.450 in Polish Red cows and 0.391 in Polish White-Backed cows in the present study, as well as the better suitability of the milk of these breeds for cheese production, demonstrated by Litwińczuk et al. [70], Teter et al. [71,72], and Wolanciuk et al. [73], may indicate an interaction between genetic traits, environmental factors, and the composition and quality of milk. The local cattle breeds analysed in the present study are in the vast majority of cases kept on small extensive farms, using feeding and housing systems referred to as traditional; moreover, these cows are not particular about feed and are distinguished by adaptation to harsh conditions and good health [16].

Due to the intensive selection of dairy cattle for the quantity of milk production, the AA and AB genotypes are predominant in highly productive breeds. The literature indicates that the A allele is associated with higher lactation yield [74,75]. The present study showed the highest milk yield in Polish White-Backed cows with the A allele in the genotype of the *CSN3* gene. This was not observed in the Polish Red breed. Sitkowska et al. [81] reported the highest yield of milk (6414.48 kg), fat (271.38 kg), and protein (209.12 kg) in Polish Holstein-Friesian cows with the AA genotype, in comparison to AB (6089.18 kg) and BB (6398.36 kg). In the Serbian Holstein-Friesian breed [82], cows with the BB genotype had the lowest milk yield (8260.00 kg), in comparison to AB (8724.73 kg) and AA (8582.72 kg), while the highest fat content (3.27%) was associated with the BB genotype and the highest protein content (3.19%) with the AA genotype. Similar relationships were reported by Bugeac et al. [83] for the Montbéliarde breed and by Hristov et al. [84] for the Bulgarian Rhodopean breed.

The frequency of individual allelic forms and genotypes is closely linked to the degree of heterozygosity, which is similar in the case of both proteins analysed in the two oldest Polish cattle breeds, at about 45%. Similar results for heterozygosity were reported by Miluchová et al. [36] in Slovak breeds in *CSN2* and by Hohmann et al. [61] in German breeds in *CSN2* and *CSN3*. Contrasting results for *CSN2* were presented by Kumar et al. [85] for the Indian cattle breed Tharparkar (H_O_ = 0.090 and H_E_ = 0.076). This comparison reveals pronounced differences between *B. taurus* and *B. indicus* cattle.

Many authors [18,19,21,23,33,74] have shown a significant relationship between the genetic variant *CSN3* B and higher concentrations of κ-CN in milk, better coagulation properties, and increased whey expulsion in comparison with the A variant, as well as links between variant *CSN2* A1 with higher milk production.

In the 1990s, it was already observed [86,87] that instead of estimating the influence of separate casein protein genotypes, it is much more useful to examine composite genotypes (*CSN2-CSN3*). Studies by Comin et al. [74], Vallas et al. [69], and Kyselová et al. [62] have shown that in the Holstein-Friesian breed, cows with at least one B allele in the composite *CSN2-CSN3* genotype produced milk with the best clotting time and curd firmness. In the present study, the A1/A2-A/B genotype was the most frequent in the Polish Red breed. Comin et al. [74] and Vallas et al. [69] also observed a link between the A2/A2-A/A genotype and the yield of milk and protein. In the present study, the frequency of this genotype was higher in Polish White-Backed cows. In our search for links with dairy production, we can refer to results published by the Polish Federation of Cattle Breeders and Dairy Farmers [9]. They show that in 2023 the country’s population of Polish White-Backed cows had a higher average milk yield over 305-day lactation (4104 kg) than the Polish Red breed (3561 kg). Polish White-Backed had also higher protein yield (136 kg) compared to the Polish Red (121 kg).

## 4. Materials and Methods

### 4.1. Material Collection

The material for the study consisted of hair bulbs from 777 Polish White-Backed cows and 1000 Polish Red cows protected by a genetic resource conservation programme, collected for the purposes of breeding work. Polish Red cattle are currently bred in southern and northern Poland. At the end of 2023, the population of this breed covered by the genetic resource conservation programme numbered 4236, among which 2639 cows were subject to milk performance assessments [88]. Polish White-Backed cattle are distributed all over Poland but are particularly concentrated in the east. At the end of 2023, the number of cows of this breed covered by the genetic resource conservation programme was 1123, of which 794 were used for dairy purposes [88]. Females used only for dairy purposes were used in the present study. Among the four local breeds kept in Poland, Polish White-Backed and Polish Red are considered to be the oldest, an indigenous breed which has always been raised in this country. The criteria for inclusion in the study included the absence of genes of other breeds and at least one completed lactation; in addition, in the case of the Polish Red breed, only cows kept in southeast Poland (the original region of this breed) were used. According to data from the evaluation of the milk performance of cows in Poland in 2023 [9], the average milk yield of Polish White-Backed cows for the lactation period was 3922 kg and the yield of Polish Red cows was 3345 kg.

DNA was isolated from the samples of hair bulbs using a commercial kit for the isolation of nucleic acids from various types of biological materials (A&A Biotechnology, Gdańsk, Poland), according to the procedure described by the manufacturer. The DNA samples were stored at −20 °C until analysis.

### 4.2. Genotyping

Initially, 1960 animals were selected for genotyping, but three samples were eliminated from further analysis because they had a call rate of <0.90. Polymorphism of proteins *CSN2* and *CSN2* was determined using Illumina Infinium XT SNP technology (Illumina, San Diego, CA, USA). For the present study, the following arrays were used: EuroGenomics_MD_v2_POL, EuroGenomics_MD_v3_POL, EuroGenomics_MD_v4_POL, and EuroGenomics_MD_v4-1_POL. Beadchips were scanned in the Illumina iScan system, and the scans were analysed using GenomeStudio Software V2011.1 version 1.9.4 (Illumina, San Diego, CA, USA). All samples from one type of array were regrouped together before export using a specific cluster file created from all samples. *CSN2* and *CSN3* were analysed using appropriate probes: *CSN2_7* for *CSN2* and *CSN3_AY380228_13124* and *CSN3_AY380228_13068* for *CSN3* (Table 8).

### 4.3. Milk Production Parameters

The milk performance parameters of the Polish Red and Polish White-Backed cows selected for the study were determined on the basis of milk performance assessment results collected in the ICT database of the Polish Federation of Cattle Breeders and Dairy Farmers. The yield of milk (kg) and the yield and content of the most important milk constituents, i.e., fat (kg; %), protein (kg; %), casein (kg; %), lactose (kg; %), and dry matter (kg; %), were determined for the first 305-day or shorter complete lactation.

### 4.4. Statistical Analysis

The statistical analysis was carried out solely on data with a genotyping call rate of at least 0.95. To determine the polymorphism of the *CSN2* and *CSN3* genes in Polish White-Backed and Polish Red cattle, a statistical analysis of the results was performed using POPGENE (version 1.32). The frequency of alleles and genotypes at individual loci, the observed and effective number of alleles, the degree of observed (Het_O_) and expected (Het_E_) heterozygosity, and the inbreeding coefficient (Fixation index—Fis) were analysed [43]. In addition, Fit (total inbreeding estimate) and Fst (population differentiation) were determined. The degree of genetic variation between breeds was determined on the basis of the Fst value, according to Wright’s scale (1978): FST < 0.05—very low or no variation between populations; 0.05–0.15—low genetic variation; 0.15–0.25—moderate variation; and >0.25—high variation between populations. Hardy–Weinberg equilibrium in the cattle populations was tested by the chi square test (χ^2^) [89]. The Bonferroni correction was applied to obtain significance thresholds when the *p*-value was smaller than 0.05/N, where N is the total number of markers, equal to 3, which gives 0.02.

In addition, composite *CSN2-CSN3* genotypes were compared between the two breeds. Linkage disequilibrium (LD) was measured as r2, which is the squared correlation of the alleles at two loci [90]:

$$r^{2}=\frac{{\left[f\left(AB\right)-f\left(A\right)f\left(B\right)\right]}^{2}}{f\left(A\right)f\left(a\right)f\left(B\right)f\left(b\right)}$$

where f(AB), f(A), f(a), f(B), and f(b) are observed frequencies of haplotype AB and of alleles A, a, B, and b, respectively.

Haplotype frequencies were calculated using the EM algorithm as implemented in R package SNPassoc version: 2.1-2 [91].

### 4.5. Ethics Statement

No ethical approval was required for this study. Hair samples were collected by the Polish Federation of Cattle Breeders and Dairy Farmers as a part of the standard genotyping of breeding animals. In accordance with Resolution No. 13/2016 of the National Ethics Committee for Animal Experiments (Poland) of 17 June 2016, the consent of the Ethics Committee is not required for the collection of animal material for genotyping. In addition, the animals’ owners gave their consent to include their animals in the research.

## 5. Conclusions

In the dairy industry, there has recently been an increase in interest in genetic variants of casein proteins, particularly beta-casein and kappa-casein. This is associated with the trend of promoting A2 milk, which according to research conducted all over the world may be beneficial for human health, and with the acquisition of raw milk, whose physicochemical parameters make it possible to obtain high yields of high-quality cheese. The study showed that Polish White-Backed cows may be more predisposed to produce A2 milk, due to the high frequency of the A2 allele in *CSN2*. In addition, different *CSN2* and *CSN3* genotypes in this breed were shown to be associated with milk production traits. Milk from the Polish Red breed, on the other hand, had a higher frequency of the B allele in *CSN3*, which seems to be more favourable for cheese yield. However, just as the intensive selection for milk yield carried out in commercial breeds resulted in a high level of inbreeding, the same could occur if too much attention is paid to the presence of the A2 allele in *CSN2* in local breeds. Moreover, local breeds have much lower milk yield than international breeds such as Holstein-Friesian and will undoubtedly never achieve such a high level of production, whereas their milk has better physicochemical properties, which are of importance in cheese production. Therefore, we may ask whether the selection of animals of local breeds, carried out by breeders tempted by higher profits, will take away their specific identity, given that they are a valuable reservoir of genes which in the future may play an important role in ensuring food security and provide a source of high-quality products? To obtain a satisfactory answer to this question, research should be continued to determine how the polymorphism of casein proteins in the Polish Red and Polish White-Backed breeds is linked to milk performance traits and the technological parameters of the milk.

## Figures and Tables

**Table 1:** Frequency of *CSN2* and *CSN3* alleles in Polish Red and Polish White-Backed cows.

Breed	*CSN2*		χ^2^ d.f. = 1	*p*-Value	*CSN3*			χ^2^ d.f. = 1	*p*-Value
	A1	A2			A	B	E		
PR	0.570	0.430	0.00	0.98	0.635	0.350	0.015	1.98	0.58
PWB	0.388	0.612	1.90	0.17	0.669	0.313	0.018	5.84	0.12

Note: PR—Polish Red cattle; PWB—Polish White-Backed cattle; χ^2^—Hardy–Weinberg equilibrium (HWE).

**Table 2:** Genetic indices of the Polish Red and Polish White-Backed populations.

Parameter	*CSN2*		*CSN3*	
	PR	PWB	PR	PWB
Na	2	2	3	3
Ne	1.962	1.904	1.900	1.831
H_E_	0.490	0.452	0.479	0.425
H_O_	0.490	0.475	0.474	0.454
Fis	0.000	0.049	−0.011	0.064
Fit	0.000	0.049	−0.011	0.064
Fst	0.000	0.000	0.000	0.000

Note: PR—Polish Red cattle; PWB—Polish White-Backed cattle; Na—observed number of alleles; Ne—effective number of alleles; H_O_—observed heterozygosity; H_E_—expected heterozygosity; Fis—Wright’s [43] fixation index; Fit—total inbreeding estimate; Fst—measurement of population differentiation.

**Table 3:** Haplotype frequency in the Polish Red and Polish White-Backed breeds.

Haplotype	*CSN2_7*	*CSN3_AY380228_13068*	*CSN3_AY380228_13124*	Frequency	
				PR	PWB
1	A1	A	A	0.201	0.196
2	A1	A	E	0.000	0.000
3	A1	B	A	0.354	0.177
4	A1	B	E	0.015	0.017
5	A2	A	A	0.149	0.115
6	A2	B	A	0.281	0.495
7	A2	B	E	0.000	0.000

**Table 4:** Pairwise LD between SNPs in the population of Polish Red and Polish White-Backed cattle.

			PR	
		*CSN2_7*	*CSN3_AY380228_13068*	*CSN3_AY380228_13124*
	*CSN2_7*		<0.01	0.01
PWB	*CSN3_AY380228_13068*	0.11		0.01
	*CSN3_AY380228_13124*	0.02	0.01	

Note: PR—Polish Red cattle; PWB—Polish White-Backed cattle.

**Table 5:** Frequency of *CSN2* and *CSN3* genotypes in Polish Red and Polish White-Backed cows.

	*CSN2*			*CSN3*					
Breed	A1A1	A1A2	A2A2	AA	AB	BB	AE	BE	EE
PR	0.325	0.490	0.185	0.399	0.450	0.122	0.022	0.007	-
PWB	0.162	0.452	0.386	0.462	0.391	0.112	0.023	0.010	0.002

Note: PR—Polish Red cattle; PWB—Polish White-Backed cattle.

**Table 6:** Milk production traits in Polish Red cows of different *CSN2* and *CSN3* genotypes in first lactation.

Gene	Genotype	N	Milk (kg)	Fat (kg)	Fat (%)	Protein (kg)	Protein (%)	Casein (kg)	Casein (%)	Lactose (kg)	Lactose (%)	Dry Matter (kg)	Dry Matter (%)	Length of Lactation (day)
All		1000	3535.16 +/− 886.61	154.13 +/− 41.26	4.37 +/− 0.50	117.34 +/− 29.14	3.33 +/− 0.22	91.99 +/− 22.99	2.61 +/− 0.19	168.31 +/− 42.96	4.76 +/− 0.17	463.58 +/− 116.27	13.13 +/− 0.64	294.93 +/− 19.04
*CSN2*	A1A1	325	3498.57 +/− 839.53	153.26 +/− 39.29	4.39 +/− 0.51	115.85 +/− 27.29	3.32 +/− 0.22	90.86 +/− 21.46	2.60 +/− 0.19	166.53 +/− 40.94	4.76 +/− 0.17	458.77 +/− 110.37	13.12 +/− 0.65	293.83 +/− 20.40
	A1A2	490	3578.19 +/− 911.14	155.64 +/− 41.53	4.36 +/− 0.49	118.63 +/− 29.93	3.32 +/− 0.21	92.99 +/− 23.62	2.61 +/− 0.19	170.33 +/− 43.98	4.76 +/− 0.17	468.85 +/− 118.36	13.12 +/− 0.64	295.18 +/− 18.97
	A2A2	185	3485.30 +/− 900.06	151.64 +/− 43.87	4.35 +/− 0.48	116.51 +/− 30.17	3.35 +/− 0.22	91.35 +/− 23.89	2.63 +/− 0.20	166.08 +/− 43.64	4.76 +/− 0.18	458.07 +/− 120.67	13.14 +/− 0.63	296.22 +/− 16.60
*CSN3*	AA	399	3599.97 +/− 815.88	157.72 +/− 37.36	4.40 +/− 0.50	119.50 +/− 26.90	3.33 +/− 0.21	93.77 +/− 21.21	2.61 +/− 0.19	171.35 +/− 39.15	4.76 +/− 0.16	472.57 +/− 105.37	13.15 +/− 0.65	296.62 +/− 17.00
	AB	450	3475.62 +/− 900.40	150.76 +/− 42.73	4.34 +/− 0.50	115.47 +/− 29.57	3.33 +/− 0.20	90.52 +/− 23.35	2.61 +/− 0.18	165.26 +/− 43.87	4.75 +/− 0.18	455.20 +/− 119.37	13.10 +/− 0.62	293.79 +/− 20.68
	BB	122	3450.35 +/− 983.01	150.39 +/− 44.00	4.37 +/− 0.46	114.67 +/− 32.29	3.33 +/− 0.27	89.65 +/− 25.40	2.61 +/− 0.24	165.03 +/− 48.55	4.77 +/− 0.17	453.16 +/− 128.80	13.15 +/− 0.70	294.07 +/− 18.32
	AE	22	3961.91 +/− 1033.55	174.50 +/− 44.86	4.41 +/− 0.44	130.68 +/− 33.08	3.31 +/− 0.19	102.46 +/− 26.29	2.59 +/− 0.18	189.68 +/− 46.55	4.80 +/− 0.18	520.50 +/− 129.09	13.17 +/− 0.60	295.45 +/− 13.86
	BE	7	3842.67 +/− 1242.05	168.83 +/− 70.32	4.29 +/− 0.53	119.17 +/− 44.83	3.06 +/− 0.22	93.75 +/− 34.95	2.41 +/− 0.18	183.17 +/− 58.77	4.77 +/− 0.18	497.83 +/− 181.95	12.81 +/− 0.73	284.67 +/− 38.69

Note: no significant differences at *p* ≤ 0.01 were detected.

**Table 7:** Milk production traits in Polish White-Backed cows of different *CSN2* and *CSN3* genotypes in first lactation.

Gene	Genotype	N	Milk (kg)	Fat (kg)	Fat (%)	Protein (kg)	Protein (%)	Casein (kg)	Casein (%)	Lactose (kg)	Lactose (%)	Dry Matter (kg)	Dry Matter (%)	Length of Lactation (day)
All		777	3938.61 +/− 1080.23	160.42 +/− 48.64	4.07 +/− 0.50	130.35 +/− 37.57	3.31 +/− 0.26	101.76 +/− 29.65	2.58 +/− 0.22	185.59 +/− 52.64	4.71 +/− 0.22	504.68 +/− 143.37	12.80 +/− 0.72	289.97 +/− 25.44
*CSN2*	A1A1	126	3481.66 ^A^ +/− 1002.80	139.99 ^A^ +/− 44.98	4.01 +/− 0.47	115.55 ^A^ +/− 36.13	3.31 +/− 0.28	90.50 ^A^ +/− 28.99	2.59 +/− 0.23	164.42 ^A^ +/− 49.64	4.71 +/− 0.21	444.47 ^A^ +/− 135.33	12.73 +/− 0.73	290.95 +/− 25.08
	A1A2	351	3975.45 ^B^ +/− 1094.78	162.44 ^B^ +/− 48.89	4.09 +/− 0.51	131.53 ^B^ +/− 37.54	3.31 +/− 0.27	102.55 ^B^ +/− 29.51	2.58 +/− 0.22	187.18 ^B^ +/− 53.43	4.70 +/− 0.23	509.70 ^B^ +/− 145.01	12.82 +/− 0.71	290.82 +/− 25.07
	A2A2	300	4093.43 ^B^ +/− 1044.70	166.91 ^B^ +/− 47.67	4.07 +/− 0.51	135.37 ^B^ +/− 36.70	3.30 +/− 0.25	105.72 ^B^ +/− 28.98	2.58 +/− 0.21	192.90 ^B^ +/− 50.74	4.71 +/− 0.22	524.88 ^B^ +/− 138.19	12.81 +/− 0.72	288.55 +/− 26.02
*CSN3*	AA	359	4212.09 ^A^ +/− 1039.19	171.77 ^A^ +/− 48.54	4.07 +/− 0.52	138.86 ^A^ +/− 36.01	3.30 +/− 0.26	108.30 ^A^ +/− 28.34	2.57 +/− 0.21	198.28 ^A^ +/− 50.42	4.70 +/− 0.21	539.04 ^A^ +/− 138.64	12.78 +/− 0.74	290.80 +/− 24.37
	AB	304	3799.35 ^B^ +/− 1059.76	154.42 ^B^ +/− 45.92	4.07 +/− 0.47	126.02 ^B^ +/− 36.34	3.32 +/− 0.26	98.37 ^B^ +/− 28.79	2.59 +/− 0.21	179.07 ^B^ +/− 52.46	4.70 +/− 0.23	487.41 ^B^ +/− 139.62	12.82 +/− 0.65	290.63 +/− 24.89
	BB	87	3399.33 ^B^ +/− 996.78	139.00 ^B^ +/− 48.24	4.07 +/− 0.57	113.96 ^B^ +/− 40.55	3.32 +/− 0.31	89.62 ^B^ +/− 32.54	2.61 +/− 0.26	160.82 ^B^ +/− 48.38	4.73 +/− 0.23	437.10 ^B^ +/− 140.73	12.80 +/− 0.85	283.51 +/− 32.12
	AE	18	3798.17 +/− 1017.81	154.83 +/− 48.09	4.05 +/− 0.042	124.72 +/− 34.20	3.29 +/− 0.20	96.91 +/− 26.59	2.56 +/− 0.17	178.83 +/− 48.72	4.71 +/− 0.18	484.50 +/− 135.25	12.75 +/− 0.68	290.17 +/− 19.48
	BE	7	3317.71 +/− 1501.18	134.00 +/− 45.28	4.18 +/− 0.62	111.71 +/− 42.89	3.44 +/− 0.23	86.83 +/− 32.93	2.67 +/− 0.19	157.29 +/− 72.28	4.73 +/− 0.17	424.86 +/− 170.37	13.00 +/− 0.90	302.00 +/− 5.13
	EE	2	3953.50 +/− 358.50	154.00 +/− 15.00	3.88 +/− 0.14	121.50 +/− 12.50	3.07 +/− 0.11	94.50 +/− 10.50	2.39 +/− 0.11	190.50 +/− 17.50	4.81 +/− 0.14	439.50 +/− 45.50	12.46 +/− 0.36	259.50 +/− 13.50

Note: A, B—significant differences at *p* ≤ 0.01.

**Table 8:** Details of the single nucleotide polymorphisms (SNPs) analysed in the SNP array.

Gene ^1^	Chromosome	Position ^2^	Milk Protein Variants
*CSN2_7*	6	85451298	A1; A2
*CSN3_AY380228_13124*	6	85656792	A; E
*CSN3_AY380228_13068*	6	85656736	A; B

Note: ^1^ ID of the SNP as indicated in the EuroGenomics MD chip; ^2^ nucleotide position on *Bos taurus* chromosome.

## Data Availability

The original contributions presented in this study are included in the article. Further inquiries can be directed to the corresponding author.

## Abbreviations

The following abbreviations are used in this manuscript:

*CSN2*	Beta-casein
*CSN3*	Kappa-casein
Fis	Fixation index
HetO	Observed heterozygosity
HetE	Expected heterozygosity
LD	Linkage disequilibrium
